# Leisure-time physical activity, cardiorespiratory fitness and feelings of hopelessness in men

**DOI:** 10.1186/1471-2458-9-204

**Published:** 2009-06-25

**Authors:** Maarit Valtonen, David E Laaksonen, Jari Laukkanen, Tommi Tolmunen, Rainer Rauramaa, Heimo Viinamäki, Jussi Kauhanen, Timo Lakka, Leo Niskanen

**Affiliations:** 1 Department of Medicine, Central Finland Central Hospital, Keskussairaalantie 19, 40620 Jyväskylä, Finland; 2 Department of Medicine, Kuopio University Hospital, PL 1777, 70211 Kuopio, Finland; 3 Institute of Biomedicine, Department of Physiology, University of Kuopio, PL 1627, 70211 Kuopio, Finland; 4 School of Public Health and Clinical Nutrition, University of Kuopio, PL 1627, 70211 Kuopio, Finland; 5 Department of Psychiatry, Kuopio University Hospital, PL 1777, 70211 Kuopio, Finland; 6 Kuopio Research Institute of Exercise Medicine, Haapaniementie 16, 70100 Kuopio, Finland

## Abstract

**Background:**

Leisure-time physical activity (LTPA) and cardiorespiratory fitness contribute to mental health. Hopelessness has been linked to impaired mental health, cardiovascular events and mortality. Previous studies have focused on physical exercise and depression. We examined the associations of LTPA and cardiorespiratory fitness with feelings of hopelessness.

**Methods:**

In this cross-sectional study leisure-time physical activity, maximal oxygen uptake (VO_2max_), hopelessness and cardiovascular risk factors were assessed in a population-based cohort of 2428 men aged 42 – 60 years old at baseline.

**Results:**

Men feeling more hopeless about their future and reaching goals were less physically active, less fit and had a higher prevalence of many cardiovascular risk factors than men with lower levels of hopelessness. In a logistic regression model adjusted for age, smoking, alcohol consumption, cardiovascular disease and socioeconomic status, men engaging in less than 60 min/week of moderate-to-vigorous LTPA were 37% (95% CI 11 – 67%) more likely to feel hopeless than those engaging in at least 2.5 h/wk of LTPA. After further adjusting for elevated depressive symptoms the association of LTPA and hopelessness remained significant. VO_2max _was also associated with hopelessness, but not after adjustment for depressive symptoms.

**Conclusion:**

Moderate and vigorous LTPA and cardiorespiratory fitness were inversely associated with hopelessness in these middle-aged men. These findings suggest that physical inactivity and poor cardiorespiratory fitness is an important associate of hopelessness, a distinct element of low subjective well-being.

## Background

Hope is an important component of physical and psychological well-being [1]. Hopelessness is associated with dissatisfaction with life, depression and suicidality [2]. Previous studies have shown that hopelessness is a predictor of cardiovascular morbidity [3-6] and mortality [7], independently of depression and other confounding factors. Hopelessness is also associated with the incidence of hypertension, myocardial infarction, and cardiovascular mortality and with accelerated progression of carotid atherosclerosis in middle-aged men [4-6]. Even though hopelessness is a rather prevalent condition in general population [8] and is inversely associated with health [3-7], the mechanisms underlying this phenomenon remain unclear.

Leisure-time physical activity (LTPA) and cardiorespiratory fitness seem to protect against chronic diseases such as the metabolic syndrome [9,10], type 2 diabetes [11] and cardiovascular disease (CVD) [12,13]. Moreover, physically active lifestyle may improve mental health [14]. Previous studies have mainly focused on exercise and depression. Both cross-sectional [15-18] and prospective studies [18-21] have shown conflicting results on the association between physical activity and depression. For example, Tolmunen and colleagues [15] reported that cardiorespiratory fitness is inversely related to elevated depressive symptoms in middle-aged Eastern Finnish men. However, physical activity, rather than cardiorespiratory fitness, has been associated with better mental health and mood [16]. Mechanisms behind these associations are unclear. Confounding factors related to depression such as an unhealthy lifestyle and several chronic diseases must also be taken into consideration in assessing the relationship of physical exercise and psychological well-being.

The associations of physical activity and cardiorespiratory fitness with hopelessness have not been reported earlier. We therefore investigated the associations of LTPA and cardiorespiratory fitness with hopelessness in 2428 middle-aged Finnish men.

## Methods

The Kuopio Ischemic Heart Disease Risk Factor Study (KIHD) is a population-based cohort study in a sample of middle-aged men in Eastern Finland. Baseline data were collected between 1984 and 1989 from 2682 male participants aged 42 – 60 years old.

The Research Ethics Committee of the University of Kuopio approved the study. All study subjects gave their written informed consent. This study includes 2428 men who had complete data on physical activity, VO_2max_and depressive symptoms.

### Assessment of LTPA

The validated KIHD 12-month LTPA Questionnaire was used as described previously [13,22]. This is a detailed quantitative questionnaire assessing the duration, frequency and mean intensity of the most common lifestyle and structured LTPA of middle-aged Finnish men as recalled over the previous 12 months. Low-intensity LTPA was defined as <4.5 metabolic equivalents (METs). One MET is defined as metabolic expenditure at rest, corresponding to an oxygen uptake of 3.5 ml O_2_/kg. Cut-off of ≥ 4.5 METs for at least moderate LTPA included brisk walking, skiing, jogging, bicycling, ball games and forestry. Vigorous LTPA was defined as ≥ 7.5 METs. The durations of LTPA were calculated in min/week.

### Assessment of cardiorespiratory fitness

A graded symptom-limited exercise test was performed on an electrically braked cycle ergometer. VO_2max _was measure directly with breath-by-breath respiratory gas-exchange analysis as previously described [13].

### Assessment of hopelessness and depressive symptoms

The psychological questionnaires included two items that measured hopelessness [4-6]. These items were "the future seems to be hopeless, and I cannot believe that things are changing for the better" and "I feel that it is impossible to reach the goal I would like to strive for". Participant responded using 5-point scale (0 = absolutely agree, 1 = somewhat agree, 2 = cannot say, 3 = somewhat disagree, or 4 = absolutely disagree). Hopelessness score, with a range of 0 to 8, was created by reverse-coding and summing the items.

We used the Human Population Laboratory Depression Scale (HPL Scale) to assess depressive symptoms. The HPL Scale is a self-administered 18-item depression score that was specifically developed for screening general population samples [23,24]. A cut-off ≥ 5 was used previously to classify men with elevated depressive symptoms [24].

### Assessment of features related to the metabolic syndrome, diabetes and cardiovascular disease

Body mass index (BMI), waist circumference and blood pressure were measured as previously described [9].

Fasting blood glucose was measured using a glucose dehydrogenase method. Diabetes was defined as fasting blood glucose concentration ≥ 6.1 mmol/L or a clinical diagnosis of diabetes [25]. Serum insulin was determined with a Novo Biolabs radioimmunoassay kit. The cholesterol contents of lipoprotein fractions and serum triglycerides were measured enzymatically. Fibrinogen was measured based on the clotting of diluted plasma with excess thrombin. Serum C-reactive protein (hCRP) was measured with a high-sensitivity immunometric assay [26].

### Definition of the metabolic syndrome

The National Cholesterol Education Program (NCEP) criteria were used (for men, three or more of the following: fasting blood glucose levels ≥ 5.6 mmol/l, triglycerides ≥ 1.7 mmol/l, HDL cholesterol <1.0 mmol/l, blood pressure ≥ 130/85 mmHg, waist girth >102 cm) [27,28].

### Other assessments

Medical history, socioeconomic status, smoking habits and alcohol consumption were assessed with questionnaires [9,29].

### Statistical Analysis

Participants were categorized by tertiles based on their scores for hopelessness. Differences between men in the highest third of hopelessness and men in the lower thirds were assessed with one-way ANOVA, and where indicated, the chi-squared test. LTPA and VO_2max _were categorized by tertiles for logistic regression analyses. The associations of LTPA and VO_2max _with hopelessness were estimated using logistic regression models adjusting for covariates. Durations of LTPA (in min/week) and triglyceride, insulin and hCRP concentrations are presented as medians (interquartile ranges); other data are presented as means ± or simple percentages. Triglyceride and insulin concentrations were corrected for skewing using log transformation for statistical analysis but are presented using untransformed values. Statistical significance was considered to be P < 0.05. All statistical analyses were performed with SPSS 11.0 for Windows (Chicago, IL).

## Results

### Baseline clinical characteristics

Men with increased hopelessness had more pronounced features of the metabolic syndrome and inflammation (Table 1). Moreover, men in highest third of hopelessness were less physically active and had a lower VO_2max_.

**Table 1:** Characteristics of the 2428 middle-aged men according to tertiles of hopelessness.

	Low	Middle	High	***P ***value*
N	757	803	868	
Age (years)	51.8 (5.6)	53.0 (5.0)	54.0 (4.5)	< 0.001
Body mass index (kg/m^2^)	26.6 (3.3)	26.8 (3.5)	27.1 (3.8)	0.011
Waist to hip ratio	0.94 (0.06)	0.95 (0.06)	0.96 (0.06)	< 0.001
Waist girth (cm)	89.7 (9.3)	90.7 (9.7)	92.8 (10.4)	< 0.001
Systolic blood pressure (mmHg)	132.7 (16.7)	134.2 (16.7)	135.2 (17.5)	0.014
Diastolic blood pressure (mmHg)	88.3 (10.1)	88.7 (10.7)	89.2 (10.6)	0.201
Fasting blood glucose (mmol/l)	4.7 (0.8)	4.8 (1.2)	4.9 (1.4)	0.004
HDL cholesterol (mmol/l)	1.30 (0.29)	1.29 (0.30)	1.29 (0.31)	0.864
Serum triglyserides (mmol/l)	1.28 (0.77, 1.52)	1.29 (0.81, 1.61)	1.36 (0.83, 1.60)	0.090
Fasting serum insulin (mU/l)	11.1 (7.3, 13.1)	11.7 (7.4, 13.7)	12.0 (7.5, 14,0)	0.048
C-reactive protein (mg/l)	2.10 (0.64, 2.11)	2.28 (0.71, 2.35)	2.87 (0.78, 2.88)	0.001
Fibrinogen (g/l)	2.95 (0.54)	3.03 (0.57)	3.10 (0.59)	< 0.001
Total LTPA (MET hours/year)	1799 (793, 2313)	1724 (759, 2179)	1611 (607, 2074)	0.041
Total LTPA (minutes/week)	466 (223, 602)	455 (202, 591)	449 (171, 601)	0.704
LTPA < 4.5 METs (minutes/week)	274 (82, 364)	287 (80, 362)	284 (73, 385)	0.728
LTPA ≥ 4.5 METs (minutes/week)	192 (48, 250)	168 (43, 229)	165 (21, 217)	0.030
LTPA ≥ 7.5 METs (minutes/week)	56 (3, 74)	49 (0, 57)	33 (0, 35)	< 0.001
VO_2max _(ml × kg^-1 ^× min^-1^)	32.1 (8.0)	30.4 (7.7)	28.5 (7.9)	< 0.001
Alcohol consumption (g/week)	76 (7, 91)	67 (6, 83)	83 (5, 103)	0.096
Smoker (%)	26.2	21.2	36.6	< 0.001
Adult socioeconomic status	7.2(4.4)	9.3 (4.4)	11.2 (4.0)	< 0.001
Metabolic syndrome (NCEP^†^) (%)	8.8	13.4	17.6	< 0.001
Cardiovascular disease (%)	14.4	17.6	26.4	< 0.001
Diabetes (%)	5.0	7.3	6.9	0.140
Elevated depressive symptoms (points on the HPL scale)	1.1 (0, 2.0)	1.7 (0, 2.0)	2.8 (1.0, 4.0)	< 0.001

Data are displayed as means ± SD, medians (interquartile ranges), or percentages.

*ANOVA for continuous variables; chi-square for categorical variables. ^†^NCEP, National Cholesterol Education Program.

### Association of LTPA with hopelessness

After adjustment for age, men with at least moderate LTPA more than 2.5 h/week had a 41% lower risk of being hopeless than men with ≤ 60 min moderate LTPA/week (Table 2, Model 1, P < 0.001). After further adjustment for potential confounding or mediating factors (Models 2 and 3), the association remained significant.

**Table 2:** Odds ratios (95% confidence intervals) for having feelings of hopelessness according to categories of physical activity and cardiorespiratory fitness in 2428 middle-aged men.

**HOPELESSNESS (upper vs. lower tertiles)**				
	Model 1	Model 2	Model 3	Model 4
Total LTPA (min/week)				
<270 min/week	1	1	1	1
270–486 min/week	0.73 (0.59–0.90)	0.76 (0.61–0.94)	0.75 (0.60–0.95)	0.81 (0.65–1.02)
>486 min/week	0.74 (0.60–0.90)	0.73 (0.59–0.91)	0.76 (0.61–0.94)	0.78 (0.63–0.97)
p	0.002	0.002	0.009	0.022
				
	Model 1	Model 2	Model 3	Model 4
Low-intensity LTPA (<4.5 METs, min/week)				
<111 min/week	1	1	1	1
111–270 min/week	0.79 (0.64–0.98)	0.81 (0.65–1.01)	0.83 (0.66–1.05)	0.81 (0.64–1.02)
≥271 min/week	0.90 (0.74–1.10)	0.85 (0.69–1.04)	0.88 (0.71–1.10)	0.91 (0.73–1.14)
p	0.292	0.102	0.252	0.393
				
	Model 1	Model 2	Model 3	Model 4
Moderate-to-vigorous LTPA (≥ 4.5 METs, min/week)				
≤60 min/week	1	1	1	1
61–150 min/week	0.64 (0.52–0.81)	0.81 (0.64–1.02)	0.80 (0.62–1.02)	0.82 (0.64–1.04)
≥150 min/week	0.59 (0.49–0.72)	0.73 (0.60–0.90)	0.73 (0.59–0.90)	0.72 (0.58–0.89)
p	< 0.001	0.001	0.003	0.002
				
	Model 1	Model 2	Model 3	Model 4
Vigorous LTPA (≥ 7.5 METs, min/week)				
<10 min/week	1	1	1	1
10–59 min/week	0.53 (0.43–0.64)	0.67 (0.55–0.83)	0.69 (0.56–0.86)	0.67 (0.54–0.83)
≥60 min/week	0.43 (0.34–0.54)	0.61 (0.47–0.77)	0.63 (0.49–0.81)	0.65 (0.51–0.84)
p	< 0.001	< 0.001	< 0.001	< 0.001
				
	Model 1	Model 2	Model 3	Model 4
VO_2max_(ml•kg^-1^•^-1^min)				
≤28.9	1	1	1	1
29.0–35.6	0.66 (0.54–0.82)	0.75 (0.60–0.94)	0.81 (0.64–1.03)	0.87 (0.69–1.10)
≥35.7	0.53 (0.41–0.68)	0.69 (0.53–0.91)	0.74 (0.54–1.00)	0.87 (0.65–1.16)
p	< 0.001	0.003	0.037	0.242

Data are OR (95% Cl).

Model 1, adjusted for age category (logistic regression).

Model 2, adjusted for age category, smoking, alcohol consumption (abstainers and low, moderate and high intake according to g/wk consumption), cardiovascular disease and adulthood socioeconomic status.

Model 3 adjusted for Model 2 and body mass index, serum C-reactive protein and plasma fibrinogen.

Model 4 adjusted for Model 2 and elevated depressive symptoms (Human Population Laboratory Depression Scale).

The association of vigorous LTPA with hopelessness was even stronger (Table 2, Model 1, P < 0.001). Further adjustment for potential confounding factors (Models 2 and 3) did not alter the association. Total LTPA was similarly associated with hopelessness, but low-intensity exercise was not (Table 2).

In additional analyses with separate adjustment of variables in Model 2 by VO_2max_, diabetes and the metabolic syndrome, none of these potentially mediating factors weakened the association (data not shown). Adjusting for the separate components of the metabolic syndrome (fasting serum HDL, triglycerides or insulin, fasting blood glucose, waist girth and blood pressure) did not alter the association of moderate-to vigorous or vigorous LTPA with hopelessness either. Furthermore, in the association with hopelessness there was no interaction between moderate-to-vigorous LTPA and diabetes (P = 0.290), CVD (P = 0.641), or the metabolic syndrome (P = 0.225 for the interaction). There also was no interaction between high-intensity LTPA and these variables for the association with hopelessness.

**Figure 1:**
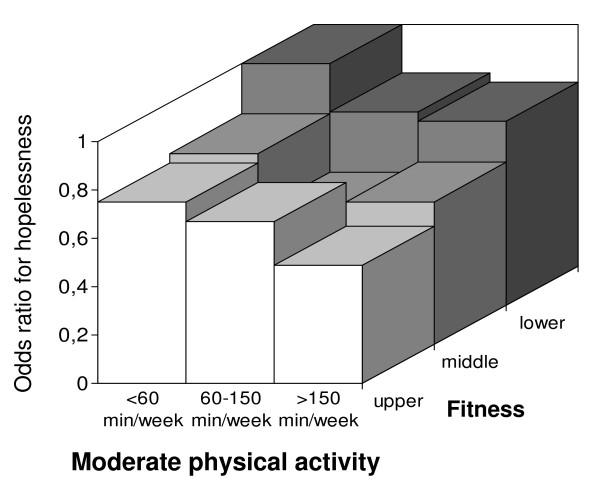
**Tertiles of hopelessness score in relation to physical fitness and at least moderate LTPA**. LTPA categories were adjusted for age, presence of CVD, adult socioeconomic status, smoking and alcohol consumption. Unfit and physically inactive men had the highest risk for hopelessness. Those men who exercised most and were fit had the lowest risk (versus the unfit and least active category, OR 0.49, 95% CI 0.33–0.73, P for the trend < 0.001). The interaction term for VO_2max _and LTPA with respect to hopelessness was not significant (P = 0.348).

We categorized the men by VO_2max _tertiles to study whether cardiorespiratory fitness modifies the association between moderate LTPA and hopelessness (Figure 1). The association seemed to be stronger in the most fit group, but the interaction term for VO_2max _and LTPA with respect to hopelessness was not significant (P = 0.348). Physically unfit and sedentary men were twice as likely to feel hopeless than fit and physically active men.

### Association of cardiorespiratory fitness with hopelessness

Men with VO_2max _over 35.7 ml • kg^-1 ^• min^-1 ^were 47% less likely to express feelings of hopeless than those with VO_2max _below 28.9. ml • kg^-1 ^• min^-1 ^after adjusting for age. After further adjustment for potentially confounding variables (models 2 and 3), cardiorespiratory fitness was still associated with hopelessness. After further adjustment separately for potential mediating factors, such as LTPA, diabetes, CVD and the metabolic syndrome, the association remained significant (data not shown). Of the components of the metabolic syndrome, only waist girth attenuated the association between VO_2max _and hopelessness significantly (data not shown). Moreover, there were no interaction between VO_2max _and diabetes (P = 0.113), CVD (P = 0.196) or the metabolic syndrome (P = 0.785 for the interaction) in the association with hopelessness.

### LTPA, hopelessness and depressive symptoms

HPL depression and hopelessness scores correlated moderately (r = 0.38, P < 0.001). The average HPL depression score in men in the highest third of hopelessness categories was higher than in the lower tertiles (Table 1). However, in the logistic regression analysis elevated depressive symptoms did not decrease the associations of moderate or vigorous LTPA and hopelessness (Table 2, Model 4, P = 0.003 and P < 0.001). Moreover, there was no interaction between LTPA and depressive symptoms in the association with hopelessness (moderate LTPA P = 0.380, vigorous LTPA P = 0.957 for the interaction). In separate analyses, depressive symptoms and LTPA were not associated.

### Cardiorespiratory fitness, hopelessness and depressive symptoms

Unlike with physical activity, after adjusting for depressive symptoms the association of cardiorespiratory fitness with hopelessness was no longer significant (Table 2, Model 4). There was no interaction between VO_2max _and depression in the association of VO_2max _with hopelessness (P = 0.109).

## Discussion

This is the first study to show that men who were physically active during their leisure-time were less likely to feel hopeless about their future and reaching goals than sedentary men. Cardiorespiratory fitness seemed to equally be accompanied by hopelessness. Moreover, these associations persisted even after adjusting for BMI, inflammatory markers and other confounding factors.

All adults are recommended to engage in at least 30 min of moderate-intensity exercise per day [30]. In our study those men exercising for at least 2.5 h per week were 27% less likely to express feelings of hopelessness than sedentary men (<60 min/week) even after adjustment for potential confounding factors, BMI and inflammatory markers. The association of vigorous LTPA with hopelessness was even stronger, reducing the likelihood of hopelessness by 37% if practiced at least one hour per week. Of importance, potential mediating factors such as VO_2max_, diabetes, the metabolic syndrome and or its components and other potential confounding factors did not attenuate these associations.

The effect of depressive symptoms on the associations of LTPA and VO_2max _with hopelessness was distinctly different. Odds ratios for total, moderate and vigorous LTPA for hopelessness were not attenuated in logistic models adjusting further for depressive symptoms, whereas the association of VO_2max _and hopelessness was no longer significant. Cardiorespiratory fitness was strongly associated with elevated depressive symptoms even after adjustment for various potential confounding and mediating factors. The relationship between LTPA and depression, however, was not found in this cohort, as reported previously [15]. This suggests that hopelessness and depression are overlapping, but distinct entities. The findings also suggest that moderate or vigorous LTPA may ameliorate or protect against feelings of hopelessness even if VO_2max _does not improve.

Although hopelessness is considered to be a part of depression, it is also quite common in the absence of depression. According to Haatainen and colleagues [8] the prevalence of hopelessness measured with the Beck Hopelessness scale, was 11.4% in a homogenous sample of Finnish adults. After excluding those with any self-reported mental disorder diagnosed or treated by a physician during the preceding year the prevalence of hopelessness was still as high as 7.8% [31]. Studies from this [4-6] and other cohorts[3] suggest that the correlation between hopelessness and depression scales is only moderate (r = 0.27 – 0.38). Moreover, these studies [3-6] suggest that the effects of feelings of hopelessness and depression can be disentangled, and that hopelessness may be a more powerful predictor of adverse cardiovascular outcome than depressive symptoms. The present findings suggest that LTPA helps to maintain an optimistic perspective on future and personal capabilities.

The distinction between hopelessness and depression could explain the difference in the associations of hopelessness with LTPA and VO_2max_. It is important to note, however, that cardiorespiratory fitness is only partly a reflection of physical activity. Although moderate and vigorous LTPA obviously has an effect on VO_2max_, it is also determined partly by genetic factors [32]. How this may relate to feelings of hopelessness and depression is still unclear.

The strength of this cross-sectional study is its large population-based design and detailed assessments of different psychosocial factors and features related to cardiovascular disease. Furthermore, VO_2max _was measured directly by a maximal symptom-limited cycle ergometer exercise test with analysis of respiratory gas exchange, an accurate and highly reproducible measure of cardioresipiratory fitness [33]. Physical activity was not assessed using physical activity monitors, but the questionnaire used to measure physical activity is valid and repeatable [22]. Hopelessness and depressive symptoms were measured using self-reported questionnaires instead of structured interviews. Although these questionnaires are relatively simple and are designed for epidemiological use rather than clinical diagnosis, they have been well validated [4,24]. Both of these scales have been widely used in the prediction of outcomes and as outcomes [4-6,15,23,24]. The HPL depression scale is significantly correlated with the Beck Depression Inventory in an outpatient population (r = 0.66) [24], and it is also similar to other symptom checklists, such as the Center for Epidemiologic Studies Depression scale [24]. However, we lack information of correlation between the HPL hopelessness questionnaire and other hopelessness scales such as Beck Hopelessness scale. The findings of this study cannot be generalized to women and men in different age and ethnic groups. Because of the cross-sectional design, we cannot draw conclusions about the directionality of the associations of physical activity and VO_2max _with hopelessness.

## Conclusion

Physically active middle-aged men are less likely to feel hopeless about their future and reaching goals than sedentary men. Cardiorespiratory fitness was also related to reduced feelings of hopelessness, but this association was partially explained by depressive symptoms. Considering that hopelessness is an important determinant of mortality, cardiovascular morbidity and low subjective well-being, this study provides additional evidence for the health benefits of physical activity and fitness. Physically active lifestyle not only helps to live a physically healthier life, but it may also improve happiness by helping to maintain a positive attitude and optimistic perspective on the future and oneself.

## Competing interests

The authors declare that they have no competing interests.

## Authors' contributions

MV participated in the design of the study, performed the statistical analysis and drafted the manuscript. DEL participated in the design of the study and helped to draft the manuscript. JL, TT, RR, HV, JK and TK have been involved in drafting the manuscript or revising it critically for important intellectual content. LN conceived of the study, and participated in its design and coordination and helped to draft the manuscript. All authors read and approved the final manuscript.

## Pre-publication history

The pre-publication history for this paper can be accessed here:


